# Phytochemical Diversity and Therapeutic Potential of *Campomanesia adamantium*

**DOI:** 10.1021/acsomega.5c06262

**Published:** 2025-10-06

**Authors:** Renata Nascimento, Matheus Antônio de Novaes da Silva, Adryan Franklin Luiz Ferreira, João Pedro Farias Pimentel, Elizabete de Souza Cândido, Vitor Brito Salentim, Octávio Luiz Franco

**Affiliations:** 1 S-Inova Biotech, Postgraduate Program in Biotechnology − Molecular Pharmacology Laboratory; 186072 Catholic University of Dom Bosco, Campo Grande 79117-900, Brazil; 2 Bachelor’s Program in Biomedicine, 186072 Catholic University of Dom Bosco, Campo Grande 79117-900, Brazil; 3 Center for Proteomic and Biochemical Analysis, Postgraduate Program in Genomic Sciences and Biotechnology, Catholic University of Brasilia, Brasília 71966-700, Brazil; 4 Bachelor of Agronomy Program, 186072 Catholic University of Dom Bosco, Campo Grande 79117-900, Brazil

## Abstract

There is a growing interest in health-promoting foods with functional properties, and alternative sources are gaining attention for their industrial potential due to sensory qualities and consumer acceptance. In that sense, the guavira *Campomanesia adamantium* is a fruit plant that has been gaining popularity in South America, especially in large cities, due to its flavor and its nutritional composition rich in minerals and bioactive compounds. The species is also used in traditional medicine due to its pharmacological properties, associated with various parts of the plant. Pharmacological data concerning its importance have been widely observed in *in vitro* and *in vivo* studies, with tumor antiproliferative, antioxidant, antihyperlipidemic, anti-inflammatory, antinociceptive, antidiarrheal, antirheumatic, antimicrobial, and photoprotective activity, and the absence of cytotoxic or toxic effect. These nutraceutical characteristics are associated with the composition of several phytochemicals, among which limonene, α-pinene, β-pinene, monoterpenes, sesquiterpenes, flavonoids, and vitamin C stand out, in addition to the presence of fibers that help improve gastrointestinal transit and absorption of water and lipids. Our study suggests that this plant really has pharmacological properties of interest; however, more extensive research is needed to establish a potential strategy, especially on the productive agronomic aspect and development of genetically improved cultivars with higher levels of substances of interest.

## Graphical abstract

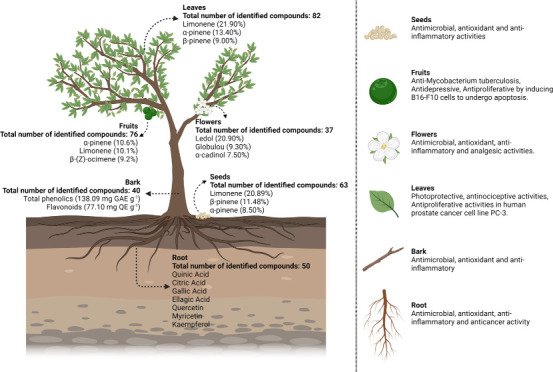

## 1 Introduction

The diversity of fruit species in regions of tropical and subtropical climates can be successfully used in several industrial segments, which can potentially aggregate value to some of these under-used crops or native species with an extractive nature. Aromatic shrubs often produce edible fruit.^1^

Many members of the Myrtaceae family have been used in folk or traditional medicine, mainly for antidiarrheal, anti-inflammatory, antimicrobial, antioxidant, antirheumatic, or depurative effects, and in the control of dyslipidemia, among other functions.^2^ Among the various species in the family, *Campomanesia adamantium* (Cambess.) O. Berg., popularly known in South America as “guavira,” “guabiroba” or “guabiroba do mato,” is endemic to the Cerrado (Neotropical Savanna) and is characterized as a spice due to its aromatic characteristics and as a raw material for industry. The species has medicinal properties in its leaves, stems, fruit, flowers, and seeds and is widely used in folk medicine.^3,4^

Guavira exploitation has been growing, especially in Brazil, occurring on an extractive scale by traditional or rural communities. Since the 2000s, the first works that arose from phytotechnical programs for morphogenetic and agronomic characterization have appeared, and more recently, studies have been carried out on the phytochemical composition and pharmacological properties.^5–7^

In traditional and indigenous medicine in Brazil, Bolivia, and Paraguay, guavira treats different diseases or disorders. The indication for use is generally related to the plant’s organs, mixed or not with other plants. The leaves and stems (peel, fragment, or scrapings), dried or in their natural state, are used in the treatment of diarrhea and urinary tract infections and for anti-inflammatory, liver, hypertension, and antirheumatic purposes.^8–10^ When used for medicinal purposes, the plant parts are dehydrated and crushed, thus obtaining a fine powder, usually used in preparations or infusions.^8,10^ The fruit can be added to sugar cane brandy, which facilitates the solubilization of various compounds in ethanol (from 35 to 50 °GL) in hot infused tea. The fruit is also used in the preparation of jellies and syrup, with subsequent use in desserts, confectionery, restaurants, etc.;^8,11^ these uses are closely related to the flavor of the plant. Like the fruit from other plant species, a wide range of compounds is present in guavira, such as the basal components (carbohydrates, lipids, proteins, dietary fibers, and vitamin C) and other phytochemicals (total phenolics, tannins, terpenoids, among others). Phytochemicals present in guavira are reported to be health-promoting compounds. The recommendation for use is related to the disease or for health promotion associated with the part of the plant to be used. This occurs due to specific molecules or classes of compounds, such as phenolic compounds.^12–14^ In this regard, designing more specific research, especially on plant metabolomics, proteomics, and genomics, is essential.

Although widely consumed, the species is not intensively cultivated. Regular production records are scarce in the literature, mainly due to how they are sold, which usually occurs in local communities, small markets or fairs, and even along highways, limiting information about the production/extractive methods and price of fruit or other plant parts. The use of native species in phytomedicine in South America faces regulatory hurdles marked by a lack of standardization, gaps in toxicology, and misalignment among national agencies. The chemical variability inherent to plants, combined with differences in soil, climate, and cultivation practices, makes it challenging to ensure batch-to-batch reproducibility, demanding sophisticated quality control methods such as chemical fingerprinting and marker validation.^15,16^ From a safety perspective, many extracts still lack robust preclinical and clinical toxicology data, which limits their acceptance in more stringent registration processes. Furthermore, the region lacks regulatory harmonization (each country adopts its own classifications and requirements for the use of herbal medicine, supplements, and traditional products), fragmenting the market and raising development costs. This scenario underscores the necessity for unified regional protocols to mitigate uncertainties and expedite safe access to products derived from native biodiversity.^17,18^

There are no commercial accessions, varieties, or cultivars, which also limits the technical data in this regard, showcasing the need for research into the phytotechnological improvement of the species. The volume of information generated for the guavira has been high in the last two decades. However, some bottlenecks remain, such as domestication, field cultivation, and elucidation of the chemical compounds present.

Guavira has morphological characteristics similar to the blueberry (*Vaccinium myrtillus* Ericaceae), which could be used as primary research to improve the species. Despite this, research into the general aspects of the species, selection of genotypes, physiological, ecophysiological, agronomic studies, and the determining factors in the quality of the final product has been carried out slowly.^19–21^ Although research is available on cultivation and medicinal properties, several new fronts of investigation still need to be studied to boost its appreciation and provide complete elucidation of its potential.

Although there is not much information regarding commercial planting and seedling production of this species, guavira has excellent potential for economic exploitation since, as mentioned previously, its leaves and fruit are highly appreciated. However, exploitation takes place in an unsustainable form of extractive cultivation, without any technical support for the management and conservation of the species.^22^

There has been a significant decrease in the guavira population in recent years.^23^ Environmental degradation threatens plant populations,^20^ often due to the constant spread of agriculture and livestock farming. However, a phylogenetic survey using molecular markers was carried out in five populations, with collections from 2011 to 2017, and correlated with the use and coverage of the period. The results presented by the authors show that the high rates of the inbreeding coefficient can, in the long term, lead to genetic depression, primarily due to the fragmentation by anthropic activities in the Cerrado, causing a population bottleneck.

Despite the growing body of research, significant methodological challenges remain. Issues such as species domestication, the establishment of large-scale cultivation protocols, and the full elucidation of its chemical compounds continue to pose major bottlenecks. The lack of genetically improved cultivars with high levels of agronomically and pharmacologically relevant substances represents a barrier to the standardization of studies and to the sustainable commercial exploitation of the plant.^24^

Given this context, the current study aimed to gather information regarding historical and botanical aspects, cultivation, uses of *C. adamantium*, contributions to the development of products of greater added value, and a compilation of the medicinal properties and biotechnological applications.

As an evaluative strategy and inclusion criteria, we used bibliometric information in several areas of knowledge and different means of dissemination in English, Portuguese, and Spanish (articles, books, bulletins, and technical communications, among others), with subsequent qualitative evaluation. The databases used were Scopus, Web of Science, Google Scholar, Pubmed, Mendeley, and Scielo. The time of publication was not used as an exclusion criterion to show aspects of the evolution of research with *C. adamantium*. The terms chosen to develop a documentary database were guavira, guabiroba, *Campomanesia adamantium*, *C. adamantium,* Cerrado guavira, cultivation, production, industrial potential, phytoconstituents, nutritional composition, phenolic compounds, flavonoid activity, antioxidant activity, essential oil, disease, antimicrobial, cytotoxic, antitumor and biotechnological potential.

A final bank of 87 scientific articles was compiled after classifying them according to different criteria based on topic, academic area, country of origin, and year of publication. A comprehensive review of other literature sources, data sources, and research papers was conducted to find and discuss various aspects of their use. For the specific topic of guavira, 52 scientific productions were used as the basis of this work. Brazil is the country that contributes the most to research, with 100% of publications. The research areas were Pharmacology and Medicine (46.80%), Botany and Agriculture (31.92%), and Food Science and Technology (21.28%). For the assembly of Table 3, the images of molecules, or the establishment of its design, the tool Explore Chemistry PubChem (National Library of Medicine) was used.

## 2 Botanical Description

*Campomanesia adamantium* is a terrestrial neotropical species with a center of origin and distribution in central Brazil, northern Paraguay, and western Bolivia (Figure 2B). shows the whole plant (Figure 1A), bark (Figure 1B), leaf (Figure 1C), flowers (Figure 1D), fruit (Figure 1E), seeds (Figure 1F), and fragments of roots for tea (Figure 1G), and the leading chemical constituents present are displayed. The popular name “guavira” is derived from the Tupi-Guarani indigenous language, meaning “bitter bark tree,” possibly attributed to the presence of tannins. This typical species usually propagates and develops in natural habitats or environments conserved by man.^19^ The dissemination of seeds in the natural environment occurs mainly by means of some mammals, and several species of birds, as it is a food that they highly appreciate.^21^

**Figure 1:**
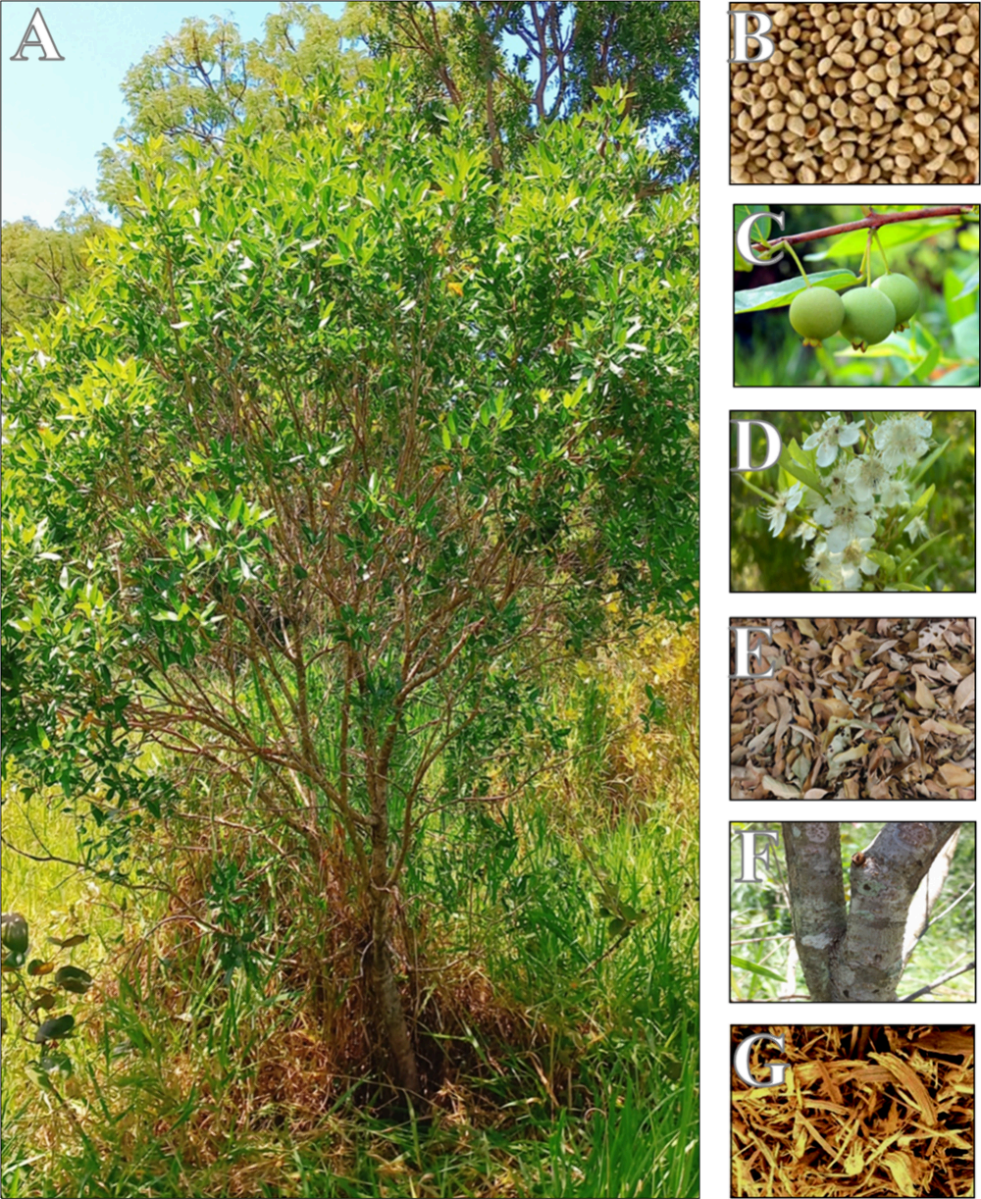
Photographs of the *Campomanesia adamantium* (Cambess.) O.Berg. plant and its morphological parts. (A) whole plant, (B) seeds, (C) fruit, (D) flowers, (E) leaves, (F) bark, and (G) fragments of roots for tea (photograph courtesy of Author: Vitor Brito Salentim. Copyright 2025).

The species belongs to the kingdom Plantae, division Magnoliophyta, class Magnoliopsida, order Myrtales, family Myrtaceae, genus *Campomanesia*, and species *C. adamantium*. Several botanical synonyms have been established for the species, from its first cataloguing until the one established today. This was due to the great genetic-morphological diversity, such as architecture and growth habit, color, and leaf size. The scientific names linked to the species are as follows: *Campomanesia caerulea* O. Berg.; *Campomanesia caerulescens* O. Berg.; *Campomanesia cambessedeana* O. Berg.; *Campomanesia campestris* (Cambess.) D. Legrand.; *Campomanesia desertorum* O. Berg.; *Campomanesia glabra* O. Berg.; *Campomanesia glareophila* Barb. Rodr. ex Chodat & Hassl.; *Campomanesia lancifolia* Barb. Rodr. ex Chodat & Hassl.; *Campomanesia microcarpa* O. Berg.; *Campomanesia obscura* O. Berg.; *Campomanesia paraguayensis* Barb. Rodr. ex Chodat & Hassl.; *Campomanesia resinosa* Barb. Rodr.; *Campomanesia vaccinioides* O. Berg.; *Psidium adamantium* Cambess.; *Psidium campestre* Cambess.

The morphological characters can be seen in Figures 1B to H. Next, the morphological descriptors of the species are pointed out. The architecture of canopy growth varies according to genetic diversity and the region in which the guavira biotype is found, generally controlled by microclimatic factors. Since it is a robust plant adapted to the Cerrado’s seasonality, guavira is easy to cultivate. However, this species has numerous varieties, with different formats of stem, canopy, height, and even fruit, which in the future would affect the harvesting and distribution of the fruit.^6,26–29^ The stem may be smooth or fissured with deep furrows or in plates. The leaves vary from 7.5 to 12 cm in length, domatia absent or present, acute, chordate or subcordate base, entire/revolved margin, developed petiole. Inflorescence in axillary position, uniflora or in raceme, auxotelic. Due to the species’ climatic seasonality and ecophysiological aspects, flowering occurs at the beginning of the spring season in South America (between September and November).

The flowers have ovate or triangular sepals, flower buds always with five lobes and five petals, bracteole or linear profile persistent until fruit formation, oblong nonapiculated anthers, and more than six ovules per locule. The flowers are pollinated by bees (*Apis* sp.).^30^

Under the environmental conditions of the Cerrado, fruiting occurs in the summer period (September to February). The fruits are meaty, berry-like, with a rounded shape, presenting green coloration when immature and variable postmaturation color (green, yellow, and orange). The number of seeds per fruit varies from one to four; they have a false, glandular, or membranous testa, a rudimentary embryo in a cotyledon with a long, curved axis. presents biometric values for the characterization, corresponding to the variation in data available in the literature. Variations in fruit size are related to genetic and edaphoclimatic conditions. These physical parameters are available in Table 1, where fruit ranging from 3 to 7 g can be seen, ranging from 16 to 23 mm in polar diameter and 16 to 25 mm transverse diameter. It is worth noting that the data presented are from collections carried out in the northern portion of the Cerrado.

**Table 1:** Guavira (*Campomanesia adamantium*) Fruit: Physical Parameters^a^

parameter	value
external color	green
pulp color	yellow-green
shape	circular
weight (g)	3–7
polar diameter (mm)	16 −23
transverse diameter (mm)	16–24
seed + peel (%)	3.71
pulp (%)	46.24

a Alves et al., 2013;^31^ Mota et al., 2021.^32^

## 3 Genetic Diversity of *Campomanesia* Genus

Within the order Myrtales, the Myrtaceae family, and specifically the tribe Myrteae, is recognized for its high species richness, with roughly 5,500 species in 144 genera found primarily in tropical and subtropical zones. A characteristic phytochemical signature of this family is the prominent production of secondary metabolites, notably terpenoids and tannins, which are consistently isolated from genera such as *Psidium*, *Myrciaria*, *Eugenia*, *Syzygium*, and *Campomanesia*.^27^ The quantitative and qualitative expression of such compounds demonstrates high metabolic plasticity, resulting from the interplay between the genotypic background and environmental pressures exerted by edaphoclimatic conditions. Such phytochemical variability poses a challenge for comparative studies, requiring controlled approaches for taxonomic and functional characterization. The biological relevance of these metabolite classes is extensively documented, with evidence of their potent antioxidant, antimicrobial, and anti-inflammatory activities.^31^

The Myrtaceae family has a pantropical distribution, with South America and Australia being the main centers of diversification and domestication. The systematic relationships in Myrtaceae are complex, making conservation initiatives difficult and compromising evolutionary modeling, yet the group is considered a model for studies. In recent years, studies have been substantially increased, mainly focused on micro and macroevolutionary assessments (distribution, phylogeny, genetics, and genomics, among others).^35^ The morphology of vegetative and reproductive characters is homogeneous, making taxonomy a tiring process, even for specialists.^35^ Although it is a well-defined group phylogenetically, the subdivisions within this clade have changed since their origin based on morphological characters. Morphological data associated with molecular data helped reorganize the group into Myrtoideae and Psiloxyloideae, with all neotropical representatives of Myrtaceae being grouped into Myrteae.^36^ Using nuclear and plastidial sequences, they were separated into groups within Myrteae, corresponding to *Plinia*, *Myrcia*, *Myrceugenia*, *Myrteola*, *Pimenta,* and *Eugenia* (Figure 2).^31,37,38^ The genus *Campomanesia* is part of the tribe Myrteae, and phylogenetic studies show monophyly relationships with the sister groups, namely *Acca, Eugenia, Ugni, Myrtus* and *Lenwebbian* (Figure 2).

**Figure 2:**
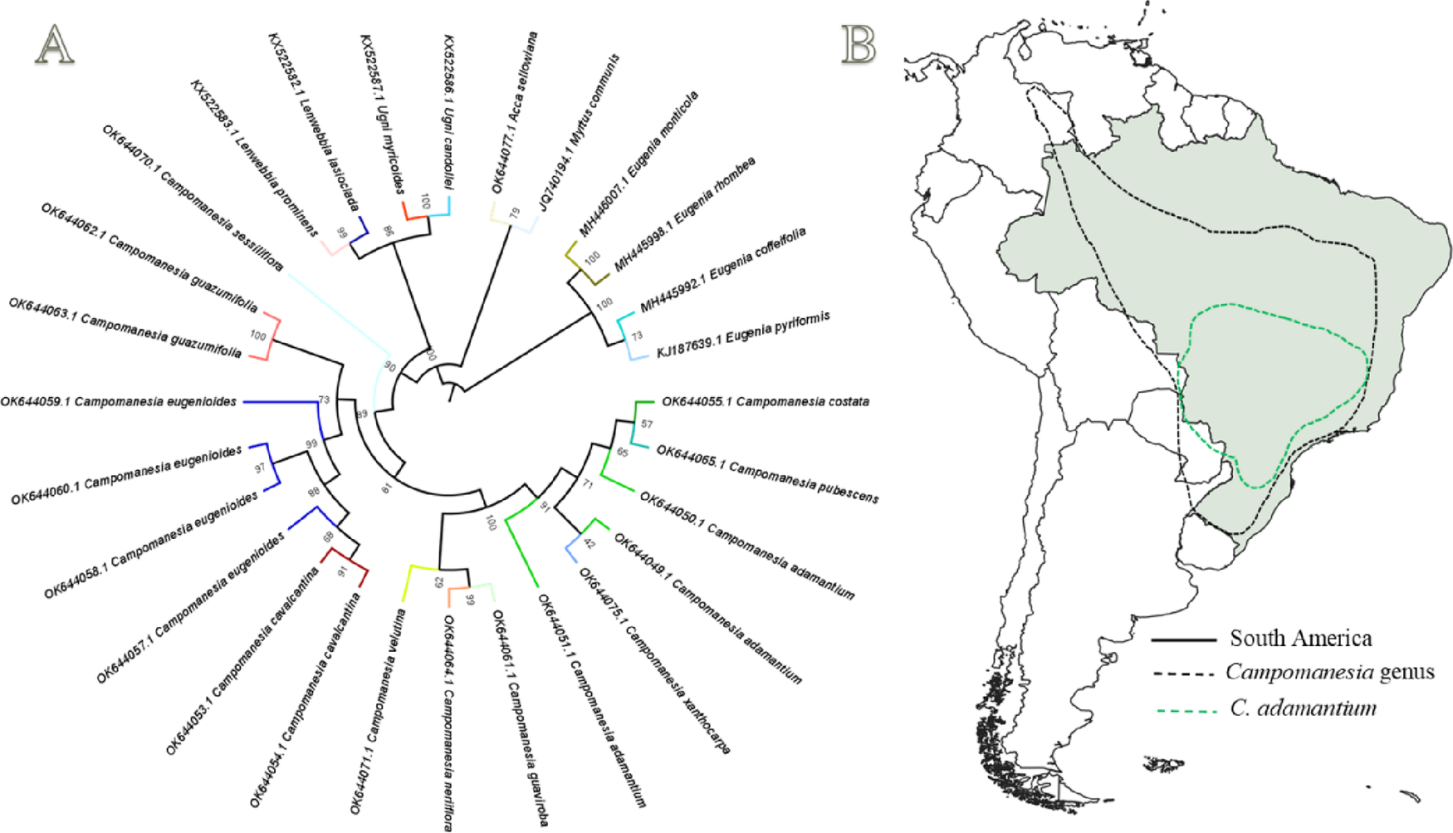
(A) Phylogenetic tree of the *Campomanesia* genus; (B) geographic distribution of the *Campomanesia* genus in South America and *C. adamantium* *.*

*Campomanesia* was first established at the end of the 18th century (Ruiz & Pavón, 1794), with many species described in the Flora Brasiliensis (Berg 1857–1859). However, the morphological description caused divergences, so many species are considered synonyms. Bentham & Hooker (1865)^39^ established the genera *Abbevillea, Acrandra, Britoa, Lacerdaea,* and *Paivaea* as heterotypic synonyms of Otto Karl Berg, and recently *Burcardia* Bellucia Neck. Ex Raf. was also included in the genus. *Campomanesia* is restricted to South America; all species are found in Brazil.^33^ In general, *Campomanesia* species are used in traditional medicine, with some studies suggesting that infusions of *C. xanthocarpa* leaves can be used to treat diabetes, control obesity, and control LDL cholesterol.^41^ *C. guazumifolia* treats liver problems.^35^ Generally, the genus species have excellent anti-inflammatory and antioxidant potential.

The genus *Campomanesia* has 36 species with a wide geographic distribution from Argentina to Mexico, and 32 species are found in South America and Brazilian territory [Figure 2B – geographic distribution), namely *C. adamantium, C. anemonea, C. aromatica, C. aurea, C. blanchetiana, C. costata, C. cucullata, C. dichotoma, C. eugenioides, C. grandiflora, C. guaviroba, C. guazumifolia, C. hirsuta, C. ilhoensis, C. laurifolia, C. lineatifolia, C. littoralis, C. lundiana, C. neriiflora, C. pabstiana, C. phaea, C. prosthecesepala, C. pubescens, C. reitziana, C. rufa, C. schlechtendaliana, C. sepalifolia, C. sessiliflora, C. simulans, C. speciosa, C. velutina e C. xanthocarpa*.^24,42^

A cluster analysis (Figure 2) shows the genetic divergence among Campomanesia species, grouping them into three Clades. Clade 1 presents two Subclades, composed of Subclade A with the species *C. costata, C. pubescens*, *C. adamantium*, *C. xantochocarpa* and Subclade B, composed of *C. guaviroba*, *C. neriiflora* and *C. velutina*. Clade 2 presents a conformation with two subclades, corresponding to Subclade A, being composed of the species *C. cavalcantina* and *C. eugenioides* and Subclade B composed of C. *guazumifolia*. Finally, Clade 3 is composed only of *C. sessiflora*. The geographic distribution data of these species do not show a relationship with genetic variability.

Scientific knowledge of plant species in their natural habitats and common uses is essential for developing genetic and environmental conservation strategies,^6^ significantly minimizing the loss of genetic resources, as with *C. indiana*. This species was endemic to Brazil but is included in the Red List of the International Union for Conservation of Nature (IUCN) as an extinct species, with only one herbarium specimen deposited in the Botanical Garden of Rio de Janeiro (register 79096 of September 10, 1952).^43^ Crispim et al. (2018)^37^ addressed a population genetic study of *C. adamantium* plants in the western Cerrado, including the territory of Brazil and Paraguay. It was found that using land for intensive agricultural and livestock practices promotes a significant decrease in genetic variability. The most studied species were C. *xanthocarpa*, C. *pubescens*, and C. *adamantium*, with the highest use reported in Nutritional composition and Phytomedicinal importance.

This species also has a wide geographical distribution, as shown in Figure 2, and it is a cultural reference in the central-south region of Brazil. Several works have tried to establish the phenotypic characteristics of the plants through morpho-descriptive characters.^33,40,45^ The genetic profile through molecular biology,^25,44^ but some gaps are perceived due to the sizable morphogenetic variation and wide distribution.

Currently, many tools are used to assess biodiversity and genetic variability. The era of OMIC technologies shows the potential of these tools in elucidating the characteristics, as mentioned earlier, with an emphasis on genomics and metabolomics.^46^ Metabolomics has become an indispensable tool for elucidating the nutraceutical and chemosystematic properties of plant species, for example, for *C. adamantium* itself, where variations between the chemical compositions of essential oils and volatile compounds of leaves and flowers are attributed to genetic and climatic factors and phenological cycle.^47^

Despite the potential and visibility for domestication and development of a commercial fruit crop, few targeted studies have been conducted for *C. adamantium*. Literature data show changes in genetic studies over the last 20 years, but still limited to genetic evaluation using conventional molecular markers.^44,48^ Molecular markers are tools that help assess genetic variability, including microsatellites (simple sequence repeats/SSRs), which were used because they are typically multiallelic, polymorphic, and have high heterozygosity.^25^ The set of microsatellites specific to *C. adamantium* and the results differed from studies of inbreeding in wild populations, indicating that the use of species-specific sets of microsatellites can provide more accurate information on genetic variability.^44^

Crispim et al. (2019)^44^ developed a panel of microsatellite markers that are useful for future studies of the genetic diversity of *C. adamantium* from several sampling points in the western part of the Cerrado. The results showed that the primers successfully amplified 36 SSR loci, in which all markers were polymorphic in the populations studied (n = 45). The alleles ranged from 2 to 14, with a mean of 8.14 per locus. Cluster analysis showed a branched tree, often found in a group of bushes of plants of the same species, with the possibility of a relationship between the sampled individuals. The species exhibits outcrossing, especially by floral visitors,^5^ which corresponds to a natural mechanism to increase genetic variation, reducing the chances of inbreeding depression and allowing a more lavish adaptation of the population to changing environmental challenges.^40^ Furthermore, reproduction is characterized by self-incompatibility, a widespread genetic mechanism to prevent self-fertilization due to the evolutionary advantages of outcrossing.^44^ The cross-fertilization present in the species could also explain the high morphological variation among its individuals and describe a possible process of hybridization among species of the genus *Campomanesia*.^33^ Moreira et al. (2022)^34^ compared the genetic diversity found in different species of *Campomanesia*, where most of the variability occurred within populations, with negligible diversification between different populations. A similar pattern has been described for *E. uniflora* (L.) (Ferreira-Ramos et al., 2008),^49^ with greater genetic diversity within populations.

A cluster analysis showed two groups with solid divergence support (89%), considering the species *Psidium guajava* as an outgroup. The first group contained two subgroups supported by a robust bootstrap (95%).^34^ The first subgroup contained the species *C. phaea, C. hirsuta*, and *C. laurifolia*. All species are widespread in the Atlantic Forest.^22^ The second subgroup contained *C. ilhoensis* and *C. guazumifolia,* species distributed in the caatinga. The second group included unknown *Campomanesia spp*., *C. xanthocarpa*, *C. adamantium, C. velutina*, and *C. pubescens*, Brazilian species found in the Brazilian Cerrado.^50^

The plastid DNA of *C. xanthocarpa* was sequenced, which showed a stable plastome structure. Furthermore, although the plastid DNA retains the same general structure as the other 47 Myrtaceae species studied, regarding the number and position of genes, it is the shortest plastid genome recorded within the family.^51^ The ycf2 gene sequenced in this study was one of the six genes with the highest polymorphism at the family and tribe level, confirming the potential of this plastid region for DNA decoding at the species level.^52^ The data obtained indicate the direct applicability of this type of study for phylogenetic research using the plastid DNA of *Campomanesia* species.

The biggest problem in the use of species is related to extractive action, and the imbalance in terms of consumption of native species in the form of extractivism and their replacement in natural environments is notorious.^53^ Here, we present a case study for the *C. adamantium* species as a strategy to mitigate barriers to its exploitation and increase its value in the face of growing demand for its use, especially as it is the most consumed and researched species. The demand for its fruits is growing year on year, and given this scenario, there is a clear need for studies aimed at preservation and sustainable use in agricultural systems.^54^ There are barriers to exploitation, especially cultivation, but various actions have been taken to map the diversity, and research efforts are being made to leverage studies on these. Another vital point for plant diversity studies is related to the phenological species parameters. There is heterogeneity in the reproductive cycle of plants, with a flowering and fruit production frame of up to four months. This is typical for sexually reproducing plants, which makes it possible to plan crosses in genetic improvement programs and increase yield in commercial crops to advance research and development of commercial genotypes of *Campomanesia adamantium* significantly.

## 4 Nutritional Composition

Few bromatological categorization studies have been carried out with the species. In this context, here we compile data from prominent publications referring to the nutritional composition of the pulp fruit. The values obtained by Vallilo et al. (2006),^55^ Alves et al. (2013),^56^ Melo (2017),^57^ and Ortega et al. (2019)^58^ are shown in Table 2.

**Table 2:** Physicochemical Constituents in Different Parts of the Guavira Plant *Campomanesia adamantium* (Cambess.) O.Berg^a^

		acidity	moisture	ash	carbohydrate	protein	fat	fiber	vitamin C
parameter	pH	g 100 g^–1^citric acid	g 100 g^–1^	g 100 g^–1^	g 100 g^–1^	g 100 g^–1^	g 100 g^–1^	g 100 g^–1^	mg 100 g^–1^
pulp	3.80–4.73	0.68 −1.20	78.90–86.35	0.33–0.98	7.64–11.60	1.06–2.26	0.55–1.50	1.640–9.00	229.00–234.00
fruit peel		0.95	63.68	0.56	5.26	3.12	3.65	19.25	218.24
residue (seed+peel)			63.70	0.74	3.0	3.17	5.33	24.05	
mineral	Na	P	K	Ca	Mg	Cu	Zn	Fe	Al

parameter	mg 100 g^–1^	mg 100 g^–1^	mg 100 g^–1^	mg 100 g^–1^	mg 100 g^–1^	mg 100 g^–1^	mg 100 g^–1^	mg 100 g^–1^	mg 100 g^–1^
pulp	0.83–21.56	17.00–170.30	121.71–130.40	8.77–19.90	10.37	0.03	0.16–0.22	1.07–1.08	0.59–1.59
seed	5.82	406.52	254.8	46.08	19.81	0.32	1.06	5.02	2.07
fruit peel	23.34	23.32	202.36	25.98	15.30	0.05	0.11	1.45	ND*
residue (seed + peel)		1.52	257.47	31.53			0.67	1.08	

a Vallilo et al., 2006;^55^ Alves et al., 2013;^56^ Lima et al., 2018;^61^ Melo, 2017;^57^ Ortega et al., 2019.^58^ *Not Detected.

The nutritional composition of *C. adamantium* varies depending on edaphoclimatic and genetic factors. In general, the soils of the Cerrado are poor in nutrients and have high acidity caused by hydrogen and aluminum, factors that interfere with plant metabolism. The macronutrients showed concentrations compatible with several other fruits from cultivated species, including carbohydrates, proteins, and lipids, resulting in a maximum caloric potential of 88.29 kcal 100 g^–1^. The pulp has significant minerals, especially potassium, phosphorus, and calcium, for micronutrients. No data were found regarding the centesimal characterization of guavira leaves and seeds. Few data were found regarding the characterization of residual material, especially fruit peel. The available data suggest that the nutritional composition is appropriate for the fruit to be used as a fiber source, in addition to having higher protein and lipid content than the pulp, as well as the content of vitamin C relative to that of the pulp (218.00 mg 100g^–1^). It is noteworthy that in addition to the chemical composition, the health aspects of the species are associated with bioactive compounds of the secondary metabolism. The fruit has an acidic flavor with a citric, persistent, and pleasant aroma.^55^

The chemical/nutritional profile of fatty acids in seeds was studied by Machate et al. (2020).^59^ The authors extracted the oil at room temperature by fixed maceration using hexane as a solvent and dried supernatant in a rotary evaporator. The oil obtained was placed in an airtight amber glass bottle and stored in a freezer at −18 °C for later analysis. As new information, the study showed that *C. adamantium* seeds are an excellent source of oil with chemical qualities and thermal stability, making it a potential edible vegetable oil for producing soaps, lotions, and biofuels. The characterized profile was oleic (34.13%), palmitic (53.02%), palmitoleic (3.97%), α-linolenic (0.10%), lignoceric (0.14%), myristic (0.20%), caproic (0.42%), stearic (2.45% and, linoleic (5.56%).

Fruit collection is related to aspects of safe food.^59^ The authors reported the levels of metal, nonmetals, and metalloids in guavira pulp according to the sampling sites (a roadside with vehicle traffic, the edges of an area with intensive modern agriculture, and a nearby forest). Regarding concentrations of heavy metals and metalloids that exceeded the Food and Agriculture Organization of the United Nations (FAO) and World Health Organization (WHO), the values were arsenic (1.96 mg 100 g^–1^), chrome (0.03 mg 100 g^–1^), cobalt (0.07 mg 100 g^–1^), lead (5.36 mg 100 g^–1^), manganese (0.05 mg 100 g^–1^), molybdenum (0.10 mg 100 g^–1^), and nickel (0.06 mg 100 g^–1^).^60^

Other components are found in guavira, such as the presence of essential oil, yellow in color, corresponding to 0.06% (v/w), which contributes to the characteristic aroma of the fruit. It is associated with monoterpenes, limonene, α-pinene, and β-pinene. Item 5 presents compound identification values and concentrations of the main constituents of essential oils, established by gas chromatography/mass spectrometry and chemical analysis. It is a well-established scientific fact that gut bacteria play a crucial role in this process. They break down large, complex molecules, such as polyphenols from plants, into more minor, simpler metabolites. These resulting metabolites are more easily absorbed into the bloodstream and are often responsible for the plant’s therapeutic effects, possessing greater bioavailability and biological activity than the original compounds.

## 5 Phytomedicinal Importance

The literature reports the correlation between the consumption of plants and the reduced risk of chronic diseases. In this topic, we address the main works on this subject, mainly focused on secondary metabolism compounds that were reported for their bioactivity, such as essential oils, total phenolic compounds, tannins, flavonoids, flavanones, chalcones and terpenoids (mono and sesquiterpenes).

Available studies on *C. adamantium* indicate low acute toxicity. Experiments in animal models demonstrated that the LD_5__0_ is greater than 2000 mg.kg^–1^ orally, with no relevant clinical signs, organ weight changes, or mortality observed.^62,63^ Studies with leaf infusions also confirmed the absence of significant toxicity in acute and subacute analyses, reinforcing the safety of the species at experimental doses.^64^ According to OECD criteria, these results classify the species as low-risk in acute toxicity models, although further investigations on chronic effects and validation in humans are still required.

Multiple studies have been performed to understand better and define the potential therapeutic uses of *C. adamantium* as an agent in treating and preventing diseases. Table 3 shows the main bioactive compounds depending on the plant organ, class, chemical structures, and functionalities.

**Table 3:**
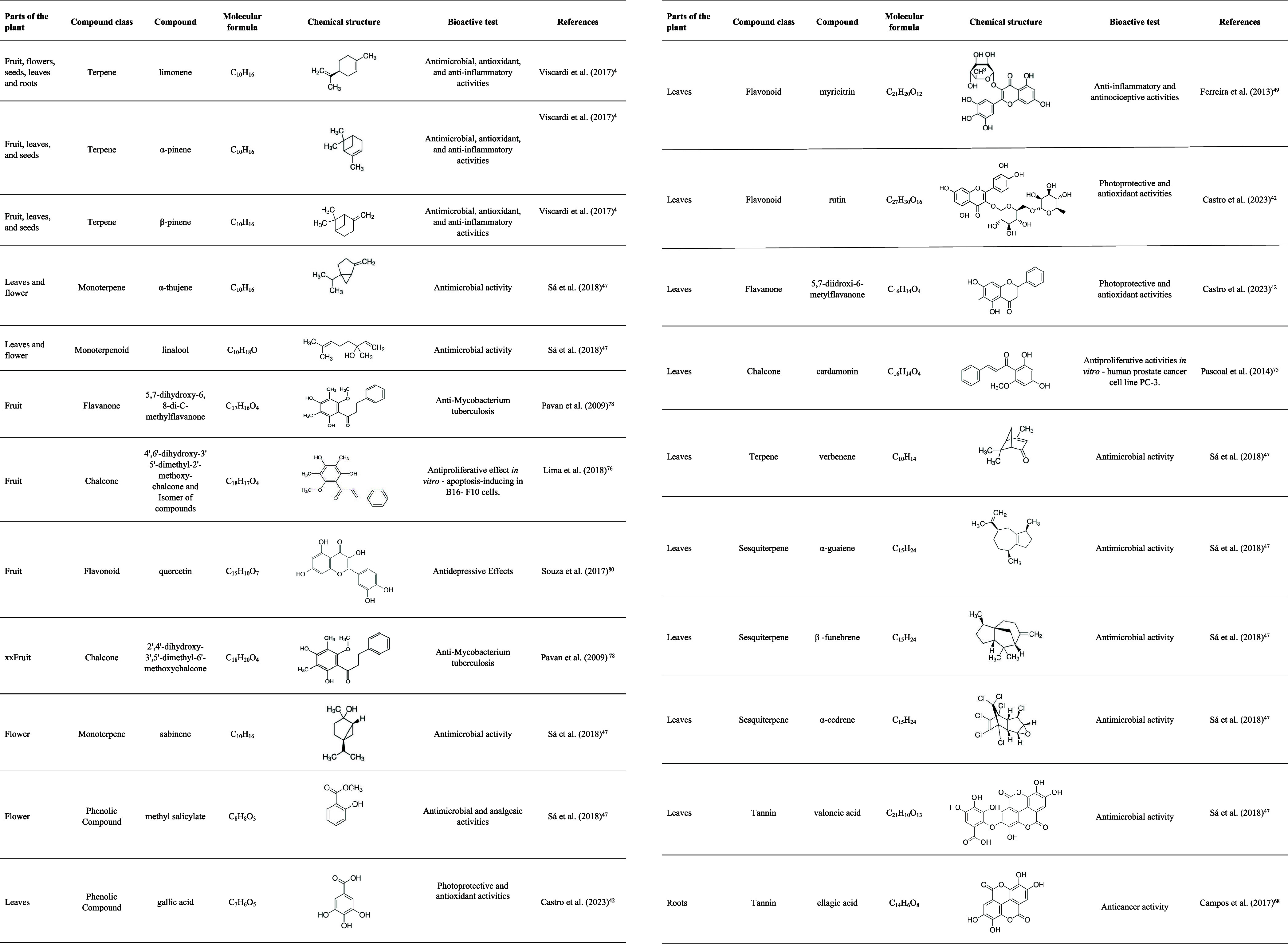
Bioactivity of Secondary Compounds in Different Parts of Guavira Plant *Campomanesia Adamantium* (Cambess.) O.Berg and Their Biological Activities *In Vitro* and *In Vi* *vo*

The administration and consumption of phytotherapeutic components may only be recommended after approved toxicological reports.^65–67^ In this regard, based on *in vitro* cytotoxicity studies in peripheral mononuclear blood cells and *in vivo* toxicity in experimental models, *C. adamantium* and its extracts (aqueous, ethanolic, and methanolic) did not cause death or clinical signs of toxicity during treatment and post-treatment periods.^8,13,57,66^ These results classify the extracts as having low acute toxicity, however further studies on chronic toxicity and potential drug interactions are still needed to establish a complete safety profile, aiming at future clinical applications.^65,66^

### 5.1 Phenolic Compounds

The seasonal factor affected the metabolism of guavira when the content of total phenolic compounds and tannins was evaluated, showing the seasonal effect of the seasons on the variation of the components, visibly separated when the authors used principal component analysis (PCA) for the evaluation. In the summer, the values were lower (3.75% and 2.91% for phenols and tannins, respectively) than in the spring (4.89% and 9.56% for phenols and tannins, respectively). This factor can influence the availability and bioactivity of the compounds.^59,70^

In this item (5.1 Phenolic compounds), we show the main secondary compounds from guavira reported in the literature, as well as the class of these compounds, their molecular and structural formula, *in vitro* or *in vivo* bioactivity, the country where the investigation was carried out and references (Table 3). Several plants are employed as sources of antioxidant action compounds, especially rich in total vitamins and phenolics. For *C. adamantium*, numerous studies have been conducted, and the antioxidant effect is attributed primarily to vitamin C, phenols, and flavonoids. It was previously shown that guavira has a seasonal variation in secondary compound contents in its leaves.^65,68,70^

The most usual method of evaluating *in vitro* antioxidant activity of free radical elimination is by DPPH (2,2-diphenyl-1-picrylhydrazyl), where half of the maximum inhibitory concentration is calculated from tested concentrations (IC_50_). Coutinho et al. (2009)^71^ report significant variability in results for compound content and antioxidant activities throughout seasons, and there is a higher concentration and consequently more significant scavenging of free radicals in spring-gathered plant material, an effect associated with the vegetable’s protection mechanism faced with the emergence of new leaves and induction of flowering.

The phenolic compound content varies between 15 and 74 mg g^–1^ GAE (gallic acid equivalent), and the elimination of DPPH IC_50_ free radicals ranges from 2648 to 3502 μmol TE g^–1^ (trolox equivalent) of ethanolic extract of leaves. The chromatographic analysis indicates the presence of flavonoids as the main constituents, including isoquercitrin and quercetin.^72^

As previously reported, the sampling performed in four subregions of the Northwest region of the Cerrado established the content of total phenolics (variation from 7.2 to 21.2 mg g^–1^), flavanones (variation from 3.49 to 30.17 mg g^–1^) and chalcones (variation from 11.24 to 194.66 mg g^–1^).^73^ All extracts showed high antioxidant activity with a wide range in the radical scavenger pathway (DPPH) range of 52.0 to 92.2% and inhibition of linoleic acid oxidation from 14.6 to 94.2%. The literature considers the high antioxidant effect of guavira extract (250 μg mL^–1^) to be due to the presence of compounds such as ellagic acid and gallic acid, obtaining a result around 15 times higher than vitamin C.^20^

Furthermore, at that time, some authors suggested that new works could be carried out to isolate and purify active components. In this context, it was established that the constituents of guavira show different responses. The method of drying the leaves (natural drying and oven drying) did not appear to strongly affect the content and quality of essential oils.^74^ The authors identified 35 and 34 compounds, respectively, of which the majoritarian constituents were spathulenol sesquiterpenes, cariofilene oxide and cermacren B. However, when evaluating antioxidant activity (DPPH), it was found that the active concentration was high, exceeding 700 μg mL^–1^. Thus, the low antioxidant activity of leaf essential oil is evidenced, a fact directly related to the low concentration of secondary metabolites capable of reacting to and neutralizing free radicals.

A bioactivity-guided study evaluated ethanolic extracts of leaves and fruit of guavira against prostate cancer cells (PC-3). The compound cardamonine (2E)-1-(2,4-dihydroxy-6-methoxyphenyl)-3-phenylprop-2-en-1-one) was isolated from leaves, and *in vitro* tests were performed. The results showed that the molecule inhibited the proliferation of prostate cancer cells and decreased the expression of the protein NF-kB, which plays a central role in inflammatory responses, regulating immune and antitumoral activity. Furthermore, an effect on inducing apoptosis in the cell line was observed by analysis by flow cytometry, showing that this compound induced DNA fragmentation.^75^

Phenolic compounds have potential anticancer activities, and in this context, researchers conducted a study of the chemical profile of dichloromethane extracts from guavira pulp and peel against in vitro antiproliferative evaluation of melanoma cells.^76^ Thirteen compounds were identified in both extracts, followed by the isolation of seven compounds, among which dimethylchalcone showed the highest antiproliferative activity with an Growth Inhibition 50% (IG50) of 7.11 μg mL^–1^. The pulp extract activated caspase-3, an essential enzyme for maintaining homeostasis and regulating apoptosis, in 29% of cells at 7.11 μg mL^–1^, and caused a 50% decrease in nitric oxide (NO) release. For this reason, the authors characterized guavira as a source of antineoplastic bioactive compounds.

The extracts were obtained using dichloromethane, and cytotoxicity was determined by assessing growth inhibition (GI_5__0_), total growth inhibition (TGI), and cytotoxic effects against B16–F10 melanoma. DEGPE exhibited superior cytotoxicity among the tumor cell lines, with pronounced potency against U-251 (GI_5__0_ = 4.89 μg mL^–^^1^; TGI = 12.77 μg mL^–^^1^), whereas DEGPU showed moderate activity (GI_5__0_ = 32 μg mL^–^^1^). At the highest concentration, DEGPE induced 88% cell death in B16–F10 cells, compared to 25% for DEGPU. *In vivo*, both extracts significantly reduced the metastatic pulmonary tumor burden, as evidenced by IR-780 fluorescence imaging and macro/microscopic analyses. The results highlight the promising anticancer activity of *C. adamantium* fruit extracts and support further investigations into their bioactive constituents.^76,77^ Regarding cytotoxicity, the reported CC_5__0_ values for different parts of the plant vary depending on the extract type and the cell line used. The ethanolic peel extract showed a CC_5__0_ of 229 μg mL^–1^ in NIH-3T3 cells, whereas the ethanolic leaf extract exhibited a CC_5__0_ of 457 μg mL^–1^ in the same cell line. Ellagic acid, as an isolated compound, showed a CC_5__0_ of 100 μg mL^–1^ in NIH-3T3 cells. The pulp extract presented a higher CC_5__0_ of 858 μg mL^–^^1^ in NIH-3T3 cells, while the methanolic leaf extract had a CC_5__0_ of 269 μg mL^–1^ in Vero cells. Overall, these data indicate that *C. adamantium* extracts exhibit low cytotoxicity in nontumor cells, with variations related to the plant organ, extraction solvent, and the cell line employed.^76–78^ The methodological challenges in studies with *C. adamantium* reflect a broader difficulty in research involving diverse plant species, including the complexity of standardizing extracts and the scarcity of clinical trials. Overcoming these bottlenecks, for both this and other plant species, depends on the adoption of reproducible protocols and the identification of active metabolites that can validate safety and efficacy in humans.

The antihyperlipidemic activity has been confirmed with the root extracts, resulting in decreased lipid peroxidation and the serum level of lipids, improving risk factors for developing cardiometabolic diseases.^20^ The researchers treated hyperlipidaemic live models daily, through gavage for 8 weeks with 200 mg of extract per kg of body mass. The results reduced total cholesterol and triglycerides, similar to normolipidaemic animals and hyperlipidaemic animals treated with simvastatin (30 mg kg^–1^ body mass) and ciprofibrate (2 mg kg^–1^ body mass). Only an increase in liver mass was noted. Nevertheless, liver enzymes remained unchanged (alanine aminotransferase and aspartate aminotransferase), and body mass and other organs were not modified using extracts.

### 5.2 Flavonoids

The chemical composition of guavira was monitored in an area along the border between Brazil and Paraguay (the Northwest portion of the Cerrado), in relation to the four seasons.^73^ The results suggested that in the summer (with high temperature and water availability), there was an increase in glycoside flavonoids and a drop in aglycone flavonoids. As for the spring (mild temperature), there was an increase in the content of flavanones and chalcones. The variation in the concentration of compounds also influenced the increase or decrease in antioxidant activity.

Flavonoids are mainly studied due to their antioxidant activity, which provides numerous health benefits. Furthermore, these compounds have been widely utilized as antimicrobial, antitumor, antiviral, antiangiogenic, and neuroprotective agents.^79–81,87^ The presence of flavonoids such as chalcones and flavonones in different plant organs has been described (Table 3). The flavonoids myricitrin, quercetin, myricetin, rutin and cardamonin are described as having antioxidant, photoprotective, antiproliferative, antimicrobial, antinociceptive and anti-inflammatory activities.^9,75,76,80^ Aqueous extracts of guavira leaves and roots showed therapeutic activity in the prevention and treatment of leukemia. Even with different chemical compositions, the main phytochemicals identified were glycosylated flavonols, ellagic acid, and its derivatives.^68^ The results showed that neither extract presented cytotoxic activity on blood mononuclear cells and promoted Jurkat cell toxicity with IC_50_ = 40 μg mL^–1^ and 80 μg mL^–1^ for leaf and root extracts, respectively. The authors posit that the changes in intracellular calcium levels and a cell cycle arrest in the S phase activity were associated with loss of mitochondrial membrane potential.

The anti-inflammatory and antinociceptive properties were evaluated using the isolated flavanols (miricintin 125 mg kg^–1^ and miricein 250 mg kg^–1^). The results in animal models demonstrated an inhibition of paw edema, reduced the time of licking in the second phase of the formalin method, and reduced the number of contortions.^9^ The results suggest that the promoted antioedematogenic effect involves various mechanisms of anti-inflammatory action, attributed to the inhibition of the production of proinflammatory cytokines, tumor necrosis factor - α (TNF-α), and nitric oxide, and increased IL-10 production by macrophages. Due to the modulation of the release of inflammatory mediators, the antinociceptive effect was demonstrated. Among the described pharmacological mechanisms are the modulation of the NF-κB pathway by cardamonin, impacting inflammatory processes; the activation of caspase-3 induced by pulp extracts and ellagic acid, which promotes cellular apoptosis; and the inhibition of pro-inflammatory cytokine production, such as TNF-α. These findings point to relevant molecular targets, although gaps remain to be explored for several of the reported effects.^75,76^

The hydroethanolic extract from fruit peel has anti-inflammatory, antihyperalgesic, and potentially antidepressant properties.^80^ The authors also indicate that its use may be considered safe, as it did not cause lethality or changes in acute and subacute behavior or toxicity. The oral anti-inflammatory activity of the extract was evaluated in carrageenan-induced pleurisy in living models in rats, with the oral treatment of 100 mg kg^–1^ during 15 days in mechanical hyperalgesia, significantly inhibited the migration, protein extravasation, and increased mobility in the forced swim test, when compared to control and finally, after 15 days of evaluation, it prevented increased sensitivity to a cold stimulus induced by spared nerve injury (SNI). Although a rheumatic anti-inflammatory action is reported in folk medicine, no related scientific data were found.

Antimicrobial resistance is considered one of the major threats for human, livestock and environmental health. There is therefore an intense search for new natural or synthetic molecules as viable alternatives in preventing and treating these infectious agents. Based on this context, Queiroz et al. (2023)^81^ carried out a systematic review of works to understand mechanisms and indication of 98 species of plants native to Brazil, among which *C. adamantium* was included due to its potential use against pathogenic microorganisms. Here we present the compilation of tests performed against 42 different types of microorganisms (bacteria, fungi, subspecies, and strains) available in the literature for this species. Data on the phytochemical composition of the plant parts used were discussed in item 5.

The activity of the fruit extracts against *Mycobacterium tuberculosis* was reported by Pavan et al. (2009).^82^ The authors associated the positive effect with a minimum inhibitory concentration (MIC) of up to 39.1 μg mL^–1^ (compounds that exhibit a MIC of 64.0 μg mL^–1^ or less are considered promising). These authors attributed the effect to six compounds (7-hydroxy-5-methoxy-6-C-methylflavanone, 5,7-dihydroxy-6-C-methylflavanone, 5,7-dihydroxy-8-Cmethylflavanone, 2 ’,4’-dihydroxy-6’-methoxyalcone), 5,7-Dihydroxy-6,8-di-C-methylflavanone, 2’,4’-dihydroxy-3′,5′-dimethyl −6’-methoxyalcone).

Standardized reporting of biological activities is crucial. In studies with *C. adamantium*, diverse active concentrations are reported, such as an IC_5__0_ range of 40–80 μg mL^–1^ for extracts against Jurkat cells. For antimicrobial activity, testing has been conducted on different microorganisms: *Candida albicans* (MIC = 5 μg mL^–1^), *Escherichia coli* (MIC = 20 μg mL^–1^), *Staphylococcus aureus*, *Pseudomonas aeruginosa* , and *Salmonella setubal*, yielding MIC values that vary from 5 to 400 μg mL^–1^. Additionally, MIC values as low as 39.1 μg mL^–1^ have been documented against *Mycobacterium tuberculosis* . Systematically presenting this data allows for consistent comparisons of potency, evaluation of dose-dependent effects, and the prioritization of promising compounds for further investigation.^7^

### 5.3 Tannins

Tannins, a class of phenolic compounds, are subdivided into condensed tannins and hydrolyzable tannins. A particular characteristic of tannins is their interaction with proteins, denaturing them, which is the basis of their astringent, reactive oxygen species-reductive, antimutagenic, antiviral, and antimicrobial properties.^57,84,85^ The tannin content in *C. adamantium* leaves ranged from 2.25 to 4.48%.^69^ The prominent tannins found in the species were ellagic acid and vanoleic acid. Ellagic acid induces apoptosis in acute myelogenous leukemia cells and is involved in caspase-3 activation.^86^ Furthermore, it also causes changes in nuclear deoxyribonucleoside triphosphate.^87^ It inhibits tumor cell proliferation by activating the TGF-β/Smad3 signaling pathway, as well as proteins involved in cell proliferation and differentiation.^76^ Valoneic acid showed antifungal activity in tests conducted by Sá et al. (2018).^40^

### 5.4 Terpenes and Essential Oils

Terpenoids, or isoprenoids, are classified based on the structural organization and number of carbons formed by the linear arrangement of isoprene.^86^ These compounds are present in the essential oils of many plants, including *C. adamantium*. Guavira leaf oil has a predominance of sesquiterpenes (59.9%) and significant amounts of monoterpenes (28.7%).^85^ The main compounds identified in the leaf essential oil were α-pinene (13.23%), β-pinene (8.99%) and limonene (22.24%).^71^ In contrast, in the flowers, the constituents found were α-thujene (8.86%), globulol (7.4%), limonene (19.33%), methyl salicylate (8.66%) and sabinene (20.45%).^47^ This composition may vary between works found in the literature, which can be explained by the oscillation of climatic factors directly interfering with the chemical composition of essential oils extracted from plants.^87^ The compounds, as mentioned earlier, have anti-inflammatory, antimicrobial, analgesic and antinociceptive activity.^47^

Essential oils have phenolics and terpenoids as their main constituents. They can be found in any part of the plant: leaves, stems, roots, seeds, flowers, and other organs. The pharmaceutical industry and research development are interested in this component’s pharmacological properties, such as antitumor, antimicrobial, anxiolytic, antioxidant, anticonvulsant, and expectorant activity, among others. This topic presents the main constituents of guavira essential oils, present in leaves, peel, seeds, and flowers, and item 6 presents the main biological activities of these compounds.

The variability in the composition of guavira essential oil in the leaf was addressed with sampling of plants in four subregions of the Northwest region of the Cerrado (sampling four different points approximately 200 km apart). The variability may be related to the adaptation factor by pollinating agents as a reproductive strategy of the plant. In addition, it may also be associated with the different altitudes and types of soil, also resulting in different yields ranging from 0.39% (subregion 1), 0.20% (subregion 2), 0.10% (subregion 3) and 0.13% (subregion 4).^73^ The authors identified 68 compounds by gas chromatography coupled to mass spectrometry (GC/MS), arranged in Table 4.

**Table 4:** Terpenoids and Essential Oils in Different Parts of the Guavira Plant *Campomanesia adamantium* (Cambess.) O.Berg[Table-fn t4fn2]

seed	fruit	flower	leaf	bark
1.8-cineole	(e)-nerolidol	(e)-nerolidol	(e)-β-ocimene	1.8-cineole
1-epi-cubenol	1,10-diepi-cubenol	aromadendrene	(z)-3-hexenyl butyrate	1-epi-cubenol
allo-aromadendrene	1-epi-cubenol	bicyclogermacrene	(z)-β-ocimene	7-epi-α-eudesmol
allohimachalol	aromadendrene	cadina-1,4-diene	1,8-cineole	allo-aromadendrene
aromadendrene	bicyclogermacrene	cis-β-guaiene	3-thujy alcohol	aromadendrene
bicyclogermacrene	cadina-1,4-diene	cis-muurola-4(14)-5-diene	borneol	bicyclogermacrene
borneol	cryptomeridiol	cyclosativene	camphene hydrate	borneol
bulnesol	epi-longipinanol	diepi-1,10-cubenol	carvone	bulnesol
camphene	epi-α-cadinol	drima-7,9(11)-diene	cis-carveol	camphene
cedrane	germacrene D	epi-1-cubenol	cis-limonene oxide	carvacrol
cubeban-11-ol s	globulol	epi-α-cadinol	cis-p-menth-2-en-1-ol	carvone
cumene	guaiol	germacrene b	cuminal	cedrane
endofenchol	limonene	germacrene d	geraniol	cubeban-11-ol s
epi-α-cadinol	linalool	globulol	isoborneol	cumene
e-β-ocimene	seychellene	guaiol	limonene	endofenchol
germacrene b	spathulenol	humulene epoxide ii	mesitylene	epi-α-cadinol
germacrene d	terpinen-4-ol	juniper camphor	methyl geranate	eudesm-7(11)-em-4-ol
guaiol	*trans*-β-guaiene	selina-3,7(11)-diene	myrcene	e-β β-ocimene
humulene epo′xi ii	viridiflorene	seychellene	myrtenol	geraniol
isoledene	viridiflorol	spathulenol	neodihydrocarvyl acetate	germacrene b
limonene	α-acorenol	α-bulnesene	nerol	germacrene d
linalool	α-cadinene	α-cadinene	o-cymene	globulol
methyl geranate	α-copaene	α-cadinol	p-cymen-8-ol	guaiol
myrcene	α-eudesmol	α-calacorene	perillal	humulene epo′xi ii
myrtenal	α-humulene	α-copaene	p-mentha-2,4(8)-diene	isoledene
o-cymene	α-muurolol	α-cubebene	terpinen-4-ol	junenol
palustrol	α-selinene	α-gurjunene	trans-carveol	limonene
perilla aldehyde	α-terpineol	α-humulene	trans-piperitol	linalool
pinene	β-caryophyllene	α-muurolol	trans-sabinol	methyl geranate
selina-3,7(11)-diene	β-eudesmol	α-ylangene	trans-sabinyl acetate	myrcene
sibirene	β-gurjunene	β -elemene	α-campholenal	myrtenal
spathulenol	β-himachalene oxide	β -selinene	α-fenchene	nerol
terpinen-4-ol	β-selinene	β-bisabolol	α-fenchol	nerolidol
terpinolene	γ-cadinene	β-caryophyllene	α-phellandrene	o-cymene
thujopsene	γ-eudesmol	β-cubebene	α-pinene	palustrol
trans-cadina-1(6),4-diene	γ-gurjunene	β-gurjunene	α-terpinen-7-al	perilla aldehyde
trans-cadina-1,4-diene	γ-muurolene	γ-cadinene	α-terpinene	selina-3,7(11)-diene
trans-carveol	δ-cadinene	γ-eudesmol	α-terpineol	sibirene
trans-muurola-3,5-diene		γ-muurolene	α-thujene	spathulenol
trans-piperitol		δ-cadinene	β-pinene	terpinen-4-ol
u-cadinene			γ-terpinene	terpinolene
u-muurolene			δ-3-carene	thujopsene
u-terpinene			δ-elemene	trans-cadina-1(6),4-diene
valerianol				trans-cadina-1,4-diene
widdra-2,4(14)-diene				trans-carveol
α-acorenol				trans-muurola-3,5-diene
α-amorphene				trans-piperitol
α-cadinene				u-cadinene
α-cadinol				u-muurolene
α-copaene				u-terpinene
α-gurjunene				valerianol
α-humulene				widdra-2,4(14)-diene
α-muurolene				z-β-ocimene
α-phellandrene				α-acorenol
α-terpinene				α-amorphene
α-terpineol				α-cadinene
α-ylangene				α-cadinol
β-copaene				α-copaene
β-elemene				α-gurjunene
β-eudesmol				α-humulene
β-guaiene				α-muurolene
β-pinene				α-muurolol
δ-amorphene				α-phellandrene
δ-cadinene				α-pinene
δ-carene				α-terpinene
δ-elemene				α-terpineol
				α-ylangene
				β-copaene
				β-cubebene
				β-elemene
				β-eudesmol
				β-guaiene
				β-pinene
				δ-amorphene
				δ-cadinene
				δ-carene
				δ-elemene

a Coutinho et al., 2008;^65^ Cardoso et al., 2009;^83^ Viscardi et al., 2017.^4^

In the investigation performed by Sá et al. (2018),^47^ essential oil extraction yielded 1.41% (using a ratio of 1:4. w/v), with yellow coloring and pleasant aroma. Thirty-seven compounds were identified, the main constituents being verbenene (13.91%), β-funebrene (12.05%), limonene (10.32%), α-guaiene (6.33%), linalool (4.91%) and spathulenol (3.86%). Among the presented works^47,52^ there is a time gap of approximately 20 years, yet only 14 compounds are found in standard (globulol, ledol, limonene, linalool, spathulenol, α-pinene, α-terpinene, α-terpineol, α-thujene, β-gurjunene, β-pinene, β-selinene, γ-cadinene, and γ-muurolene).

For the chemical profile of essential oil of guavira flowers, the literature shows a yield of 0.23% (using a ratio of 1:4 w/w), with yellow coloring and citrus aroma. Five compounds represent the majority, namely sabinene (20.45%), limonene (19.33%), α-thujene (8.86%), methyl salicylate (8.66%) and globulol (7.4%).^40^ Viscardi et al. (2017)^4^ established the chemical profile of the essential oil of the peel (of the fruit) and seeds, also reporting a low yield, corresponding to 0.32% and seed 0.98% (w/w), identifying a total of 77 compounds in the peel, with limonene (13.07%) and tujopsene (6.96%) as the main constituents, while in the seed oil 65 compounds were identified, with limonene (20.89%) and β-pinene (11.48%) found in higher concentrations.

Tests performed using essential oils from guavira leaves against *Streptococcus mitis, S. mutans, S. sanguinis, S. sobrinus* and *Bacteroides fragilis* showed prominent antimicrobial activity.^63^ Sá et al. (2018)^47^ report that the essential oil has moderate antimicrobial activity, with MICs ranging from 100 to 400 μg mL^–1^. The authors attributed the antimicrobial activity to terpenoids present in the samples. Volatile oils were also tested against *Bacillus cereus, *B. subtilis*, Listeria innocua, *L. monocytogenes*, *Micrococcus luteus*, M. roseus, Staphylococcus aureus, S. epidermidis, *Escherichia coli*, Enterobacter aerogenes, E. cloacae, *Klebsiella pneumoniae*, *P. aeruginosa*, Salmonella enterica, Salmonella* spp, **C. albicans*, Candida krusei, C. parapsilosis, C. tropicalis, *Cryptococcus neoformans*, Trichophyton mentagrophytes* and *T. rubrum*.^40^ The results show that the extracts exhibited substantial antibacterial potential by assessing antimicrobial activity, with high antibacterial (hexane fraction) and antifungal activity (aqueous fraction, concentrated aqueous fraction of tannins and valoneic acid).

## 6 Summary and Conclusions

i. This review describes the correlation of the bioactivity of phytochemical constituents of Campomanesia adamantium with health promotion, with scientifically proven activity such as tumor antiproliferative, antioxidant, antihyperlipidaemic, anti-inflammatory, antinociceptive, antidiarrheal, antirheumatic, antimicrobial, photoprotective activity and absence of cytotoxic or toxic effect.
ii. The main compounds that present bioactivities were terpenoids (limonene, α-pinene, β-pinene, α-thujene, linalool, sabinene, verbenene, α-guayene, α-cedrene, β- -funebrene), tannins (valoneic acid and ellagic acid), flavonoids (5,7-dihydroxy-6, 8-di-C-methylflavanone, quercetin, 5,7-dihydroxy-6-methylflavanone, myricitrin), phenolics (methyl salicylate and gallic acid) and chalcones (cardamonin, 2’, 4’-dihydroxy-3′,5′-dimethyl-6’-methoxychalcone, 4’,6’-dihydroxy-3′ 5′-dimethyl-2’-methoxy-chalcone and Isomer of compounds).
iii. The time and region of collection proved to be crucial factors influencing the amount and quality of phytochemical and bioactive compounds in the materials. Data compilation indicates that the spring period gives the plants higher concentrations of phytochemicals.
iv. Although different secondary metabolism compounds have antimicrobial activities, new studies should be established to assess their potential, especially using antimicrobial peptides and other components, which can potentially be a strand of studies for the species. It became clear to us that there is a need for further phytotechnical studies to better understand agricultural characteristics, selection of the genotypes with qualities of interest in the fruit and biomolecules.
v. The bioactive compounds of C. adamantium can be incorporated into innovative formulations, provided that the procedures comply with each country’s regulatory guidelines and that protection protocols are clearly defined and standardized. Proper validation ensures quality, safety, and reproducibility in potential pharmaceutical or nutraceutical applications. Research has already shown that extracts from its bark are rich in flavonoids such as quercetin and myricetin, exhibiting antioxidant properties, platelet aggregation inhibitors, and COX-1/COX-2 pathway modulation.^89^ The incorporation of these active compounds into innovative formulations, such as nanocarriers, has the potential to enhance efficacy, contribute to the creation of intellectual property, and facilitate the path to clinical validation.^89^
vi. A potential strategy for adding value to C. adamantium could focus on technological value addition to overcome the challenges of traditional phytomedicine. The focus could shift from the crude extract approach to the identification of higher-potency metabolites through ’omics’ platforms. In parallel, the production of these compounds could be explored via biotechnological routes, seeking greater standardization and scalability. The incorporation of these active compounds into innovative formulations, such as nanocarriers, has the potential to optimize efficacy, contributing to the creation of intellectual property and facilitating the path to clinical validation. The absence of clinical studies constitutes a significant gap in current knowledge, while at the same time representing a strategic opportunity for future research aimed at validating in humans the therapeutic potential already demonstrated in laboratory assays.
